# Victims and/or perpetrators? Towards an interdisciplinary dialogue on child soldiers

**DOI:** 10.1186/s12914-015-0068-5

**Published:** 2015-10-14

**Authors:** Ilse Derluyn, Wouter Vandenhole, Stephan Parmentier, Cindy Mels

**Affiliations:** Department of Social Work and Social Pedagogy & Centre for Children in Vulnerable Situations, Ghent University, H. Dunantlaan 2, 9000 Gent, Belgium; Law and Development Research Group, University of Antwerp, Venusstraat 23, 2000 Antwerp, Belgium; Leuven Institute of Criminology, University of Leuven, Hooverplein 10, 3000 Leuven, Belgium; Departamento de Psicología del Desarrollo y Educacional, Universidad Católica del Uruguay, Av. 8 de Octubre 2733, CP 11600 Montevideo, Uruguay

**Keywords:** Child soldiers, Victimhood, Perpetrator-hood, Interdisciplinarity, Children’s rights law, Transitional justice, Psychosocial studies

## Abstract

**Background:**

Worldwide, thousands of children are acting in different roles in armed groups. Whereas human rights activism and humanitarian imperatives tend to emphasize the image of child soldiers as incapable victims of adults’ abusive compulsion, this image does not fully correspond with prevailing pedagogical and jurisprudential discourses, nor does it represent all child soldiers’ own perceptions of their role. Moreover, contemporary warfare is often marked by fuzzy distinctions between perpetrators and victims. This article deepens on the question how to conceptualize the victim-perpetrator imaginary about child soldiers, starting from three disciplines, children’s rights law, psychosocial approaches and transitional justice, and then proceeding into an interdisciplinary approach.

**Discussion:**

We argue that the victim–perpetrator dichotomy in relation to child soldiers needs to be revisited, and that this can only be done successfully through a truly interdisciplinary approach. Key to this interdisciplinary dialogue is the growing awareness within all three disciplines, but admittedly only marginally within children’s rights law, that only by moving beyond the binary distinction between victim- and perpetrator-hood, the complexity of childhood soldiering can be grasped. In transitional justice, the concept of role reversal has been instructive, and in psychosocial studies, emphasis has been put on the ‘agency’ of (former) child soldiers, whereby child soldiers sometimes account on how joining the armed force or group was (partially) out of their own free will. Hence, child soldiers’ perpetrator-hood is not only part of the way child soldiers are perceived in the communities they return to, but equally of the way they see themselves. These findings plea for more contextualized approaches, including a greater participation of child soldiers, the elaboration of accountability mechanisms beyond criminal responsibility, and an intimate connection between individual, social and societal healing by paying more attention to reconciliation.

**Summary:**

This article deepens on the question how to conceptualize the victim-perpetrator imaginary about child soldiers through an interdisciplinary dialogue between children’s rights law, psychosocial approaches and transitional justice. With this interdisciplinary perspective, we intend to open up narrow disciplinary viewpoints, and contribute to more integrated approaches, beyond a binary distinction between victimhood and perpetrator-hood.

## Background

It is estimated that about 250,000 children worldwide, both boys and girls, are involved in armed groups where they act in different roles, such as soldiers, spies, cooks, porters and sex slaves [1–5]. Acknowledging this diversity, the most comprehensive and internationally endorsed definition of child soldiers can be found in the 2007 Paris Principles, drawing on the 1997 Cape Town Principles [6], and refers to *“any person below 18 years of age who is or who has been recruited or used by an armed force or armed group in any capacity, including but not limited to children, boys, and girls used as fighters, cooks, porters, messengers, spies or for sexual purposes. It does not only refer to a child who is taking or has taken a direct part in hostilities”* ([7]:7). The recruitment of children into armed forces has been hitting the headlines in politics and the media for many years, despite the fact that this group constitutes only a small part of all children and adolescents who are affected, directly and indirectly, through armed conflict on a global scale [8]. One possible explanation is that the notion of ‘child soldiers’ often defies emotional and moral senses, due to the conflicting sub notions of childhood and warfare, whereby the ‘child’ is perceived as particularly vulnerable, as opposed to the ‘soldier’ who is regarded as inherently damaging [9, 10].

The uneasiness about child soldiering can be traced back to at least two sources. First of all, in the course of history, images of childhood and attitudes towards children have significantly evolved: first, a kind of ‘indifference’ towards childhood as a separate life-period has given way to childhood as a social, educational and cultural moratorium entitled to safe development, special care and protection; and secondly, the latter notion has been challenged by views of childhood as a life-period of evolving competence and agency, requiring equal human rights that allow autonomy and social participation, as well as supplementary rights for children accommodating their vulnerability [11–15]. More generally, children have come to be viewed as rights-bearing subjects rather than objects, which engendered a range of children’s rights-based legal and policy developments that culminated in the UN Convention on the Rights of the Child [11, 15–17]. This paradigmatic shift fostered a growing awareness and problematization of child soldiering, conceptualizing it in terms of grave children’s rights violations and placing it prominently on the humanitarian and human rights agenda. These developments incited a range of international conventions enshrining the rights of children in armed conflict and outlining measures conducive to the protection of such rights [9, 13]. Moreover, humanitarian imperatives tend to emphasize the image that child soldiers are incapable victims of adults’ abusive compulsion, stripped from legal agency and without any accountability, which does not fully correspond with prevailing pedagogical and jurisprudential discourse, nor represent the broad range of child soldiers’ own perceptions of their role [9, 10, 13, 18–20].

A second source of the uneasiness about child soldiering is related to the nature of contemporary warfare, which is often marked by fuzzy distinctions between perpetrators and victims. Not only are children forcibly recruited as child soldiers and thus actively participate in the armed conflict, also the frequently ethnic nature of these conflicts, amongst others, can turn every civilian into a potential victim or a perpetrator ready to defend his/her group’s interests [21]. Such practices both increase the risk of psychological damage to all civilians [22, 23], and pose particular challenges to the recovery, rehabilitation, reintegration and reconciliation processes of all affected youth, and (former) child soldiers in particular. This thin line between being a ‘victim’ and a ‘perpetrator’, which becomes utmost clear in the situation of child soldiers, has large implications for general processes of peacebuilding [24] and transitional justice [25], both in the short and the longer run.

As the complexity of these phenomena has dramatically increased in recent years, we argue that they can no longer be analysed from one single discipline or field of study, but require a truly interdisciplinary approach. Following Ost ([26]:543), by inter-disciplinarity we mean the attempt to embark upon a ‘dialogue’ among disciplines leading to the (partial) reorganisation of theoretical frames and operational hypotheses. Unlike multi-disciplinarity and trans-disciplinarity, the essence of inter-disciplinarity lies in organising the ‘translation’ of one scientific language into the structure and the terminology of the other(s). In this contribution, we look at the victim-perpetrator dynamic in relation to child soldiers from three different disciplines or fields of study, i.e. children’s rights law, transitional justice, and psychosocial approaches. Our central research question is how to conceptualize the victim-perpetrator imaginary about child soldiers starting from these three disciplines. With this interdisciplinary perspective, we aim at opening up narrow disciplinary viewpoints, and contributing to more integrated approaches on the reintegration of former child soldiers into their communities and societies. Apart from its theoretical value in analytical terms, we also argue that an interdisciplinary perspective is indispensable from an operational point of view, as adequate policies, programmes and projects that tackle this difficult issue can only be developed by bringing together insights from different disciplines.

The insights and proposals described in this contribution are based on two types of sources: first, an extensive literature review in each of the three fields of study mentioned; and, secondly, a series of sustainable contacts with policy makers and practitioners in the fields mentioned built up throughout the years, by means of workshops, meetings and conferences.^1^

Our contribution is structured in two major steps. We start by reviewing the issue of child soldiers from three different disciplinary perspectives, children’s rights law, transitional justice and psychosocial approaches, and list the cross-cutting themes, similarities and divergences of all three perspectives (section 2). In a second step, we offer ideas to move forward in this field of study, through interdisciplinary dialogue and mutual learning, and draw out some implications for policy-making and practice (section 3).

By focussing our paper on (former) child soldiers in particular, we do not wish to discard the fact that armed conflict affects all children and adolescents, far beyond this special target group [8]. Nevertheless, the particular phenomenon of child soldering allows us to contemplate on ‘victimhood’ and ‘perpetrator-hood’, and its relation to rehabilitation, reintegration and reconciliation processes aiming at all children affected by armed conflicts, their families, communities and society. We recognize that this analysis could have benefited from the inclusion of additional perspectives, such as anthropology, political science, sociology and other disciplines, but their involvement goes beyond the scope of this contribution.

## Disciplinary perspectives on child soldiers: victims and/or perpetrators?

In this section, we explain in a mono-disciplinary fashion how each of the three disciplines under review – children’s rights law, transitional justice and psychosocial approaches—deal with the question of ‘victimhood’ or ‘perpetrator-hood’ of child soldiers. At the end of the section, we spell out the challenges that each of these three approaches are confronted with.

### Children’s rights law

The situation of children affected by armed conflict, and in particular questions regarding children’s role as ‘victim’ and/or as ‘perpetrator’, produces many challenges for the field of children’s rights law, both legal and non-legal. A 2011 special issue of *Human Rights & International Legal Discourse* has fleshed out some salient legal issues, i.e. questions that arise within the disciplinary context ([27]; on technical legal questions, see [28–30]). Here, we seek to identify the challenges related to the concept of ‘victimhood’, by examining how child soldiers are generally portrayed, and how their rehabilitation, recovery and reintegration are considered.

#### Recruitment and use of child soldiers

Children’s rights law, and other legal sub-disciplines such as international criminal law, international humanitarian law and international labour law, all tend to focus on child *soldiers* as their primary concern, rather than on children affected by war in general. Most of the standard-setting and discussion has focused even more narrowly on the recruitment and participation of children in armed conflict, and on the age limit to be applied. Whereas Art. 38 CRC (in line with international humanitarian law and criminal law) applies 15 as the minimum age for recruitment and use in hostilities, the Optional Protocol on the Involvement of Children in Armed Conflict (OPAC) applies the age of 18 (in line with international labour law), except for voluntary enlistment with state forces (with the debate being geared towards the adoption of a ‘straight 18’ approach, regardless of whether recruitment was forced or voluntary) [31]. The African Charter on the Rights and Welfare of the Child [32] is the only human rights treaty that already applies a ‘straight 18’ approach (combined reading of arts. 2 and 22). The Security Council’s work on children and armed conflict was initially confined to child recruitment too. New triggers for listing situations and for monitoring and reporting were added in 2009 and 2011, including killing and maiming of children, rape and other sexual violence against children, recurrent attacks on schools and/or hospitals, and recurrent attacks against protection persons in relation to schools and/or hospitals ([33]:883-906).

Art. 39 CRC provides for “*measures to promote physical and psychological recovery and social reintegration of a child victim*” of, inter alia, armed conflicts. Art. 6 OPAC stipulates more narrowly that *those children who were recruited or used in hostilities* in violation of the Protocol (so not just any child victim) may benefit from “*assistance for their physical and psychological recovery and their social reintegration*”. The Paris Principles do not focus exclusively on child soldiers, but reintegration is nonetheless often mentioned in combination with release and protection, or in the context of formal disarmament, demobilisation and reintegration processes (DDR).

#### Victims or perpetrators

The attention paid to the recovery and reintegration of child soldiers reflects an acknowledgement of their victimhood. Child soldiers are mainly considered as victims. However, children’s rights approaches have always oscillated between protection (what Breen [34] has called ‘paternalism’) and autonomy. That tension is not a legal one, but goes back to the underlying notion of childhood. These two schools of thought or perspectives on childhood (protection versus autonomy) have also informed the CRC. On the one hand, there is the view that children need special protection and priority care. That was the almost exclusive theme of the 1924 and 1959 Declarations on children’s rights, which should be understood in light of the two World Wars [35]. This protectionist view has been referred to as the biomedical model of childhood: ‘children as passive victims who are psychologically scarred and vulnerable’ [36]. On the other hand, there are proponents of recognising children as autonomous individuals and ‘fully-fledged beneficiaries of human rights’ [36]. In non-legal terms, reference is made to ‘children’s agency, resilience and coping mechanisms’ [36]. Following Eide ([37]:3), it may be argued that in the CRC, a balance has been struck between these two schools:

*“The CRC sees the child as an initially highly vulnerable person in need of protection, nurturing and care who under parental guidance gradually prepares for an independent life in a social setting of rights and duties when reaching eighteen.”*

The Committee on the Rights of the Child has echoed this position ([38]:§44):

*“The evolving capacities of the child (art. 5) must be taken into consideration when the child's best interests and right to be heard are at stake. The Committee has already established that the more the child knows, has experienced and understands, the more the parent, legal guardian or other persons legally responsible for him or her have to transform direction and guidance into reminders and advice, and later to an exchange on an equal footing. [footnote omitted] Similarly, as the child matures, his or her views shall have increasing weight in the assessment of his or her best interests.”*

That balance does not solve all questions, though, for it is unclear to what extent the recognition of the child soldier’s autonomy would also imply by necessity its responsibility—including the criminal responsibility. Children may be seen as perpetrators of crimes when they have reached a certain age of criminal responsibility. What that minimum age of criminal responsibility (MACR) is, remains highly uncertain under international children’s rights law. Article 40 CRC obliges states to establish a MACR, but does not specify at which age. The CRC Committee has submitted that twelve is the absolute minimum ([39]:§32). The MACR is set in domestic law, and may therefore greatly vary. At the level of international criminal prosecution before the International Criminal Court, prosecution below the age of eighteen has been completely excluded though (Art. 26 Rome Statute).

The general tendency seems to be to emphasize child soldiers’ lack of maturity and hence their vulnerability, and not to hold them criminally responsible therefore [40, 41]. To the extent that it is accepted that they should be held accountable for their actions, criminal accountability is refuted [40, 42, 43], and/or procedural safeguards are considered necessary [39]. The latter requirement seems to refer to a juvenile justice approach, which is characterized by additional safeguards as well as the establishment of a minimum age of criminal responsibility [44–46]. As the CRC Committee has put it ([39]:§31):

*“Children at or above the MACR at the time of the commission of an offence (…) but younger than 18 years (…) can be formally charged and subject to penal law procedures. But these procedures, including the final outcome, must be in full compliance with the principles and provisions of CRC as elaborated in the present general comment.”*

A minority position has argued, in an attempt to ‘reimagine child soldiers in international law’, that child soldiers have ‘circumscribed actorship’ ([47]:98):

*“I propose approaching the individual child soldier through a model of circumscribed action. A circumscribed actor has the ability to act, the ability not to act, and the ability to do other than what he or she actually had done. The effective range of these abilities, however, is delimited, bounded, and confined. Circumscribed actors exercise some discretion in navigating and mediating the constraints around them. They dispose of an enclosed space which is theirs and in which they exercise a margin of volition. The acreage of this space varies according to an ever fluctuating admixture of disposition and situation. Although encircled, circumscribed actors are not flattened. Affected by conflict, they also affect others. Threatened and harmed, they may, in turn, threaten and harm others.”*

In sum, in children’s rights law, child soldiers are predominantly considered to be victims, rather than perpetrators. This is problematic for at least two reasons. Conceptually, it overemphasizes the paradigm of vulnerability and the need for protection, at the expense of acknowledgement of agency. Above, in practice, the portrayal of child soldiers as victims often turns out to be counterproductive in reintegrating them into their communities, and in coming to terms themselves with what they have done [17–25, 47, 48]. Let us now move to a second disciplinary perspective, transitional justice.

### Transitional justice

When societies are moving away from authoritarianism to democratic forms of government or emerge from violent conflict to situations of relative peace, debates about the serious violations of human rights and the international crimes committed in the past arise relatively fast. The new elites have an interest not to deny such calls, but to deal with them in a constructive manner to avoid further conflict [49]. ‘Transitional justice’ refers to *“the study of the choices made and the quality of justice rendered when states are replacing authoritarian regimes by democratic state institutions”* ([50]:431), or in a later policy document by the United Nations to *“the full range of processes and mechanisms associated with a society’s attempts to come to terms with a legacy of large-scale past abuses, in order to ensure accountability, serve justice and achieve reconciliation”* ([51]:4). These processes and mechanisms are commonly regarded to consist of four major components: criminal prosecutions, truth commissions, victim reparation policies, and various types of institutional reforms [52]. Some of the key issues that new regimes are facing in their pursuit of justice relate to truth-seeking, accountability of offenders, victim reparation and exploring reconciliation between former enemies [49, 53]. It should be noted that the literature and policy-making on transitional justice have emerged from the many political transitions in the world in the 1980s and 1990s, and hence have centred on violations of civil and political rights and the corresponding international crimes (killings, disappearances, torture,…). Far less attention has been paid to the violations of economic, social and cultural rights, and the problems of discrimination, marginalisation and distributive justice resulting therefrom, which often created the root causes leading to violence, civil war and international conflicts. In recent years, interest has grown in transitional justice about the interplay between the first and the second generation of human rights, which could constitute the basis for a more integrated human rights approach, also for children [54].

#### Children as victims of armed conflict

In the growing literature on transitional justice, children are predominantly conceived of as victims of armed conflict. It should however be stressed that the field of transitional justice, both in theory and in practice, has paid extremely limited attention to children as independent persons distinct from their parents, their guardians or other adults. Only in recent years, some publications with a more explicit focus on children within the context of transitional justice have been drafted [8, 29, 56–58].

Transitional justice literature offers three important distinctions to denote the many faces of victimhood [59], within the broader concept of mass victimization. The latter, in the words of Fattah ([60]:412) refers to: *“victimization directed at, or affecting, not only individuals but also whole groups. In some cases the groups are very diffuse, the members have nothing or not much in common, and the group is not targeted as a specific entity. More often, however, the acts of victimization are directed against a special population”.* The first distinction is between individual victims and collective victims, the latter being the result of violent actions directed at a specific population (e.g., ethnic, ideological or religious groups) and society at large. A second distinction is between direct victims, who have suffered direct effects such as killing, abuse and detention, and indirect victims. The latter category can be defined narrowly to include only the direct victims’ family members, who experience hardship and pain as a result of the crimes committed, or more widely to encompass persons who have been traumatised as a result of having witnessed these crimes being committed, such as neighbours, friends and bystanders. The third distinction is based on the time dimension, and relates to first- and second-generation victims. According to Huyse [59], violent conflicts can produce a new generation of young people who are traumatised in various ways, and this may be a source of new conflict in the future. It is clear that children who are affected by armed conflict can be victims of many sorts, i.e. they can be victims of both the first and the second generation, they can be direct and indirect victims alike, and they can be part of individual and collective victimhood. All three distinctions are not only relevant to understanding the relationship between the harm done and the person(s) affected, but they also constitute important criteria for identifying who can participate in transitional justice mechanisms that are set up to deal with the past and construct a new future.

Although mechanisms of transitional justice developed over the last twenty years have paid some attention to children as victims of human rights violations and international crimes, they still have hardly focused on the roles children could play within such mechanisms. Examples of truth commissions that have paid attention to child victims, where children have testified about their experiences and have been able to express their expectations for the future can be counted on the fingers of one hand [58]: by way of example, the South African Truth and Reconciliation Commission held some special children’s hearings to allow their experiences to be known to the country [61]; the Sierra Leone Truth and Reconciliation Commission established a protocol with child protection agencies in the country to allow children to participate in the proceedings as witnesses; and in Liberia, a memorandum of understanding between the truth commission and the National Child Protection Network listed various strategies for the protection of children who participated in the commission’s hearings, like documenting their experiences and acknowledging their roles in the future development of the country [56]. Even lesser attention is paid to children in courts and tribunals for criminal prosecutions or civil proceedings: prosecutions of crimes committed against children remains problematic (e.g. in Colombia and the DRCongo),children’s access to judicial proceedings tends to be very limited, and special measures to protect them when they are included tend to remain exceptional [56]. On the other hand, it should be noted that the first case concluded at the International Criminal Court in The Hague related to the recruitment and conscription of child soldiers in the Eastern DRCongo, for which Thomas Lubanga was sentenced in 2012 [62]. One area of transitional justice that has arguably affected children the most concerns the systems and procedures for reparations set up in many jurisdictions [29]. Following the Van Boven/Bassiouni Principles [63], the right to reparation for victims of serious human rights violations is not limited to monetary compensation, but also includes four other categories of reparation: restitution of property and rights, rehabilitation measures (such as material and psychological assistance), satisfaction (e.g., judicial investigations, apologies, memorials,…), and guarantees of non-repetition in the future of the past violations (focused on the reform of state institutions). Moreover, children are particularly affected by the rehabilitation measures that aim to reintegrate and resocialize them into regular society after the violent conflict has ended. Some authors also suggest that children are no longer exclusively seen as passive subjects who can benefit from certain services and privileges, but that they can also be conceived of as active actors who possess interesting ideas and are able to make proposals about their own future. To take these into account when designing rehabilitation and reintegration programmes is far from an easy task, but very much in line with a ‘process-oriented approach’ to reparations [64]. An important report argues that much more work needs to be done to reform institutions in such a way that they create child-focused and child-friendly environments away from hostilities and conflict [29]. The above lines thus make clear that the large number of children affected by armed conflict pose huge challenges for transitional justice mechanisms, both in focusing on child victims of conflict, and in allowing children to participate in the proceedings of such mechanisms.

#### What about perpetrators of serious crimes?

The field of transitional justice is not only concerned with the consequences of atrocities for victims, but also aims at establishing the accountability of the perpetrators of serious crimes and human rights violations. The case of child soldiering hereby raises a particular issue, namely that these children cannot only be regarded as victims of armed conflict, but are also perpetrators of serious crimes. Often forced by militia leaders or commanders to kill or torture members of their community or even their family, the children find themselves trapped in the military logic and find it difficult to return to their communities. These situations are very good examples of the so-called ‘role reversals’ that are well-known in transitional justice, as well as in criminology and victimology, namely when victims become offenders and vice versa [65]. The fact that child soldiers cannot be held criminally responsible for their criminal acts under a certain age (cf. supra) raises serious problems in terms of accountability, one of the key issues of any transitional justice. It also creates the need to develop other than purely criminal or judicial forms of accountability for child soldiers in order for them to assume responsibility for their cruel deeds, and hence become members of society and the community again. Examples of ‘alternative’ forms of accountability can be found for example in traditional conflict resolution and justice mechanisms that include all stakeholders (victims, perpetrators, community), who discuss the background of the violence and its actors, and propose solutions (reparations, reconciliation, reintegration), sometimes by means of traditional rituals, such as ‘Mato Oput’ in Uganda [66].

### Psychosocial perspectives

#### Rehabilitation and reintegration processes

For years, humanitarian interventions for former child soldiers – mostly framed as ‘DD(R)R-programmes’ (Disarmament, Demobilisation, (Rehabilitation) and Reintegration)—predominately have included efforts to ‘repair’ these children from presumed damage caused by traumatic experiences suffered during warfare [67], particularly given the widely demonstrated high prevalence rates of symptoms of post-traumatic stress disorder (PTSD) [22, 23, 68–70], depression and anxiety [69, 71], and externalizing problems in different forms [72–74] in this group. Mainly operating in inpatient rehabilitation centres, interventions have traditionally focussed on children’s healthy recovery by means of trauma-focused counseling or group therapy, aiming to facilitate their re-adaptation and return to their families and communities [75]. These first initiatives have gradually expanded their scope, including for example vocational training activities and psycho-educative programmes [67, 76, 77]. Furthermore, interventions have increasingly involved more long-term support, including follow-up of the child and his family, even after the child´s return to the community [67, 76, 78]. Although hard scientific evidence on the outcomes of these programmes is scarce, the available intervention research shows potential for reducing symptoms of psychosocial distress in former child soldiers. Nonetheless, looking beyond psychological symptomatology, accounts remain of children and youths whose ‘rehabilitation and reintegration process’ evolves problematically, and who continue to experience difficulties in several areas (education, job, mental and physical health, social relationships, etc.) [79, 80]. As we discuss in the following, in the literature these observed difficulties have been attributed to ongoing processes of discrimination, stigmatisation and even expulsion, provoked by the child´s family and previous living environment.

#### Stigmatisation processes and the victim-perpetrator dilemma

The ongoing stigmatization of and discrimination against returned former child soldiers, as shown in many studies [72, 75, 80–82] appears to be inspired by two elements: the feelings and views of the civilian communities, and, closely related, the nature of humanitarian programming in conflict and post-conflict contexts [79]. First, stigmatization and discrimination imply that the members of the communities where former child soldiers return to, not (only) perceive these children as victims [83]. They are equally considered as perpetrators, having committed atrocities against members of their own community [79]. Secondly, this process is often aggravated by the fact that humanitarian aid and intervention agencies use categorical approaches, in which certain target groups—in particular former child soldiers—are supported, and many others are not, in an effort to effectively distribute scarce resources [78, 79, 83]. These practices shape the public notion that former child soldiers are ‘rewarded’ for the atrocities committed, and moreover, that their civilian victims are not recognized nor ‘compensated’, enhancing sentiments of injustice [79]. Consequently—and considered as a normal reaction to post-conflict − people may experience deep feelings of revenge and hatred, rendering it impossible to look at returned child soldiers (only) as victims [22, 84]. More generally, the population at large still seems to feel highly ‘victimized’, with many needs unmet, culminating in the projection of these feelings towards one of the few ‘visible causes’ of their own war trauma, being the former child soldiers.

Above, also the nature of humanitarian programming influences processes of stigmatization and discrimination on a community level. Humanitarian programmes for former child soldiers—and in particular sensitization interventions—have strongly emphasized the ‘victimhood’ of conscripted children, not only in contexts where children were obviously forcibly recruited, but also where children and youth seem to indicate that joining the armed forces was (partly) their own choice. This image of children as victims of armed conflicts and recruitment, promoted by humanitarian programmes, is grounded in two main dynamics. First, many DD(R)R-programmes—and related interventions—are framed from the ‘protection angle’ of the CRC, whereby children should be protected against the devastating impact and consequences of armed conflict, and, where needed, the necessary support and care should be provided. Second, being developed and implemented by international, Western-based non-governmental organisations, many of these programmes depart from a particular view on children and ‘childhood’, which may differ from local conceptualizations of childhood and child developmental processes. The social sciences have for long debated the premises of ‘childhood’, and the cross-cultural validity of particular age limits [9]. It is widely recognized that transitions to adulthood may differ across cultures, as other indicators than age (e.g., sexual activity, economic independency, rites of passage) tend to demarcate childhood [10]. Important individual differences too make it difficult to draw a universal age limit between ‘children’ and ‘adults’. Moreover, ‘childhood’ itself is generally differentiated into several developmental stages (e.g., early infancy, middle childhood, adolescence), which are often related to biological, cognitive, social and emotional changes in children’s development. This raises questions whether, for example, adolescents are able (cognitively, emotionally, socially,…) to judge the consequences of their choices, such as joining armed forces or groups or taking part in acts of violence. On the other hand, recent studies have emphasized the ‘agency’ of (former) child soldiers [20, 5]. By giving them ‘voice’ (through interviews and other ‘participative’ methodologies), it has been demonstrated how these recruited youths themselves often stress having joined the armed force or group out of their own free will.

### Conclusions on mono-disciplinary perspectives: limitations

In the following paragraphs and by way of conclusion on the mono-disciplinary perspectives, we will highlight some of the commonalities and differences of the three disciplinary perspectives on the ‘victimhood’—and ‘perpetrator-hood’ of child soldiers, and in particular point out some of the discipline’s limitations.

#### Children’s rights law

Under children’s rights law, child soldiers are predominantly considered as victims if recruited and used in hostilities under the legally accepted minimum age. Children may be seen as perpetrators of crimes if they have reached the minimum age of criminal responsibility (MACR), but the MACR greatly varies across countries. However, notwithstanding the acknowledgment of victimhood, little attention is paid to addressing that victimization; rather, children’s rights law focuses primarily on the prohibition of recruitment and use in hostilities.

Children’s rights law keeps facing difficult questions, for which it does not seem to find an answer within its own discipline. It seems to have difficulties in addressing the role reversal that transitional justice brings to bear. If child soldiers’ autonomy is emphasised, does that imply that they are to be held (criminally) responsible for their acts, and lose their victim status? Or alternatively, does not holding them criminally responsible come at the price of downplaying their autonomy, and of stressing their vulnerability and need for protection? Moreover, an emphasis on the autonomy of the child soldier in the context of armed conflict risks having individuating and de-contextualising effects. The impression may be created that a child has the full range of options, and therefore freely decides whether to become a child soldier. Many have pointed out that even so-called ‘voluntary’ recruitment is so much determined by the context and circumstances, that there is in fact very little free choice involved. Finally, children’s rights law fails to strike a balance between ‘victimhood’ and ‘perpetrator’-hood: below the MACR, it exclusively acknowledges victimhood; above the MACR, it resorts to criminal accountability, and thereby exclusively emphasizes perpetrator-hood. Whereas it has been argued that accountability does not always imply criminal responsibility [41, 42], it remains utterly unclear what these alternatives could look like.

In sum, children’s rights law thus tend to rely on binary models (victim–perpetrator; below or above MACR; and child-adult) [85], thereby ignoring evidence from the psychosocial field and transitional justice that child soldiers are both victims and perpetrators.

#### Transitional justice

The dominant approach in transitional justice is also to view children as victims of the armed conflict. Truth commissions that analyse the human rights violations and crimes committed invariably focus on the forcible recruitment of child soldiers, and the harsh conditions they serve outside of their choice, and point at the root causes of violent conflict and militarised societies. When proposing recommendations on reparations for victims, child soldiers tend to figure among those groups eligible for reparations as direct victims of the first generation. The same approach is found in national or international criminal courts that are expected to judge those who have committed international crimes, including the forcible recruitment of child soldiers. Former child soldiers are called as witnesses to report on their experiences and provide evidence of the crimes committed. In formalised procedures of this nature, very little attention is paid to child soldiers as perpetrators of heinous crimes by having killed, tortured and maimed during their period as a child soldier, sometimes even their own family members.

Here is one of the main challenges for transitional justice, and the mechanisms dealing with the crimes of the past: how is it possible to conceive of child soldiers as possessing this ‘double face’, as victims and as perpetrators alike? Victimology and criminology have become aware over the years of the mechanism of ‘role-reversal’ that may take place, whereby victims become tired of being harassed or treated in harsh ways and turn into offenders of similar acts, thus turning their own aggressors into victims. Potentially, this process can take place several times, thus blurring the boundaries between victims and perpetrators in the longer run. While these insights stem from ordinary or classical crimes outside of the political context of massive violence, it could be argued that they also bear relevance for the case of child soldiers. In fact, psychosocial approaches teach us that children also possess a degree of agency, albeit possibly lower than adults, and that they can be held accountable for their acts commensurate to their active involvement in activities of any sort; all this within a general context of ‘vulnerable offenders’ and geared towards avoiding secondary victimisation or re-traumatisation. However, because international children’s rights prohibit any criminal accountability under the age of 18, and definitely under 16, it would be very difficult to install some form of criminal accountability for child soldiers. But maybe other forms of accountability could be envisaged, not only to confront child soldiers with their deeds but also to allow their appropriate reintegration into communities and society at large. Examples may include traditional conflict resolution mechanisms—outside and separated of criminal justice systems—that provide a forum to discuss the past, to listen to victim experiences, and to allow offenders to assume responsibility for their acts.

Another challenge to transitional justice is to know whether former child soldiers, if also considered perpetrators, are still eligible for reparations as victims and under which conditions? Providing reparations to child soldiers who are not only perpetrators but also victims would constitute a major innovation in (inter)national law in periods of transitional or post-conflict justice.

#### Psychosocial perspectives

Starting from a particular developmental view on children, and framed within the CRC (in particular the protection and provision of rights), psychosocial perspectives have put an overarching emphasis on the fact that former child soldiers are victims, and the ‘perpetrators’ part’ has been rarely considered in humanitarian programming and interventions aiming the rehabilitation and reintegration of these children. Also, in DDR(R)-programmes and other psychosocial interventions, remarkably little elements of reconciliation have been included, despite ongoing processes of stigmatization pointing at the hypothesis that the families and communities where these children are returning to (also) see them as perpetrators. By denying this connection between the individual and the social realm in the aftermath of conflicts, there is little space for successful reintegration processes—going beyond the short-term reduction of psychological symptomatology—and the hereto-connected necessary rebuilding of the social and communal networks.

## Towards an inter-disciplinary dialogue: Beyond the binary distinction between victim- and perpetrator-hood

We have demonstrated that the complexity of the phenomenon of child soldiers cannot be analysed from one single discipline. The previous section was devoted to an in-depth analysis of three (mono-)disciplinary viewpoints on victim- and perpetrator-hood of child soldiers, and showed the limitations of each disciplinary perspective on its own. In what follows, we offer some building blocks for an interdisciplinary dialogue by flagging three learning points, which highlight some of the strengths of each discipline. Key to this interdisciplinary dialogue is the growing awareness within all three disciplines, but admittedly only marginally within children’s rights law, that only by moving beyond the binary distinction between victimhood and perpetrator-hood, the complexity of childhood soldiering can be better grasped. In transitional justice, the concept of role reversal has been instructive: child soldiers may be both perpetrators and victims. In psychosocial studies, emphasis has been put on the ‘agency’ of (former) child soldiers, and it has become clear how child soldiers themselves often stress that joining the armed force or group was (partially) out of their own free will. Hence, child soldiers’ perpetrator-hood is not only part of the way child soldiers are perceived in the communities they return to, but equally of the way they see themselves. These findings plea for more contextualized approaches, which try to understand the nuanced and complex realities of young people’s entry into fighting forces, including underlying root causes, as well as local views on the motives to persist or to abandon practices of child soldiering [10, 86]. Drawing on these central points, we hereafter discuss three points opening up future perspectives.

### Children’s right to participation

Children’s rights, in particular also in their legal articulation in the CRC, have put and continue to keep children in their own right on the agenda. Whereas in an earlier period, it may have been important to simply make the point that children do have rights, nowadays we may have to develop more sophisticated arguments on which rights they have, and how these rights relate to each other and to the rights of others. In particular, it would be highly beneficial to clarify that children’s rights are a smart mix of so-called provision, protection and participation rights.^2^ In addition to establishing the right mix between these three types of rights, the importance of participation rights deserves further recognition. Participation of children may well go beyond their involvement in concrete programming issues, to fundamental processes of rethinking the accountability and responsibility of children for the acts committed and the reconciliation processes that need to take place. An open-minded perspective, in which local conceptualisations and individual and contextual differences can play a role, is thereby a prerequisite.

### Alternative ways of holding child soldiers accountable

Transitional justice is home to a variety of non-judicial and non-legal mechanisms that address the human rights violations and international crimes committed during periods of armed struggle. The key question is to conceptualise new ways of holding former child soldiers accountable for the crimes or violations they have committed or been involved in. At this stage of international law, and given the strict requirements as to the age of criminal responsibility, it is hard to imagine how international and even national criminal tribunals or courts could indict former child soldiers. For this reason, it seems more fruitful to think about other, non-judicial and non-criminal, institutions and procedures of transitional justice that allow more flexibility. Firstly, truth commissions can promote some forms of non-judicial accountability, although they are foremost focused on unearthing concrete facts and sketching the patterns of human rights violations and international crimes. This can be organised by naming names of alleged perpetrators in the final report (although seldom done in practice), or inviting individuals and organisations to come forward and explain their actions of the past (as was the case in South Africa). Also, in the case of the South African truth commission, the Amnesty Committee had the competence to award amnesties to applicants, under certain conditions. It is thinkable to use such formats for former child soldiers, the more so because truth commissions sometimes provide a space to children, thus far mostly as victims of crimes and violations (e.g., Sierra Leone). A second type of non-judicial forms of accountability relates to community dialogues, sometimes established in the context of a truth commission (e.g., East Timor) or as a free-standing mechanism (e.g., Uganda) to reflect on the past, the suffering of victims and the accountability of offenders [87]. In both countries mentioned, the community meetings have taken the form of ‘traditional conflict resolution’ sessions, whereby all participants—victims and perpetrators—in the end engage in reconciliation, and use traditional rituals to be reintegrated in society. Such procedures not only allow community members to know who was responsible for (some of) the atrocities, but also enable offenders to assume active responsibility and become full-fledged members of society, and even create the conditions for reparations to victims. Such mechanisms also offer ample space to take the material, medical and psychological needs of children duly into account and provide the necessary support hereto. Finally, a third type of non-criminal accountability can be found outside the field of violent conflict and transitional justice, namely in a genuine juvenile justice approach for ordinary crimes, as existing in several countries worldwide. Such systems acknowledge that minors may be perpetrators of criminal offenses, but are also sensitive to seeing these youngsters as victims of their personal and social environments. That is why many juvenile justice systems also provide special safeguards (such as a specific justice system for children; specific attention for effective and child-sensitive participation; and in relation to sentencing, in particular in the case of deprivation of liberty) [43] and allow for diversion measures. Moreover, the experiences of juvenile justice systems may also inspire and inform transitional justice mechanisms to introduce novel procedures and forms of sensitivity towards the needs of children.

### Connecting individual and social healing

Children’s rights law and psychosocial studies tend to ignore the importance of reconciliation, the importance of which has been documented in transitional justice. Individual healing seems indispensably connected to social healing and to the re-establishment of broader social and community networks that are often disrupted or even destroyed by the impact of warfare (and the use of civilians in war strategies, including child soldiers). The families, communities and societies where former child soldiers return to often have changed considerably following prolonged armed conflict. The terminology used in this field is striking, with terms such as *‘re’*-habilitation and *‘re’*-integration, implying that life could be turned back to ‘normal’ as it was before conflict erupted [88]. Individual rehabilitation and reintegration processes should therefore be connected thoroughly with efforts to rebuild social networks [77]. Furthermore, interventions on an individual and social level may need to be connected to developments and initiatives on the wider national (state) level, such as peace processes, amnesty acts and transitional justice processes. Rebuilding communities and societies after protracted armed conflict therefore seems to require—as the transitional justice field clearly demonstrates—in-depth processes of reconciliation, also when children are involved as perpetrators (even when forcibly recruited).

### Implications for policy and practice

In the previous sub-section, we have flagged how interdisciplinary encounters may assist in better grasping the complex victimhood and perpetrator-hood dynamics that characterizes child soldiers. Children’s rights can make sure that sufficient attention is paid to children on their own, in particular by drawing attention to the importance of participation. Psychosocial studies have revealed the complex victim-perpetrator dynamic in the self-perception of child soldiers and in the way their families and communities see them. Transitional justice has emphasized the reconciliation dimension (social and societal), and offers inspiration for dealing with the perpetrator side outside a criminal accountability logic. What do these research findings imply for policy and practice?

First of all, the global humanitarian agenda may have to move away from the excessive focus on *recruitment* of children, and pay more attention to issues of rehabilitation, reintegration and reconciliation, in an integrated and comprehensive way. It also seems untenable to continue to emphasize unilaterally the victimhood of child soldiers, as for example has been done in the work of the Special Representative of the Secretary-General of the UN and in the lobby work of many children’s rights organizations.

Secondly, a ‘categorical’ approach that targets ‘the most vulnerable’ groups, such as child soldiers, is questionable. More coherent and coordinated interventions seem needed, beyond a categorical approach, and attention is needed to build robust, mainstream systems of different types of care to support these interventions.

Thirdly, processes of rehabilitation, reintegration and reconciliation may have to extend their scope beyond the individual recovery of affected youth, in order to include also the recovery of communities and entire societies, with as ultimate goal a long-term peace building process with preventive capacities towards a new resurgence of the conflict. What may be needed here is an approach that simultaneously addresses individual, community and societal aspects of rehabilitation and reintegration. Hereby, one should carefully address the complex elements of children’s involvement with armed groups, including possible voluntary involvement, and the complex victim-perpetrator dynamics. Also, reconciliation may have to become an integral part of interventions aiming at the rehabilitation and reintegration of former child soldiers. Inevitably, this will require more attention being paid to root causes of conflict, as well as long-term commitments not only through humanitarian, but also development assistance.

Finally, the interdisciplinary dialogue strongly emphasizes the importance of making sure that children affected by armed conflict themselves are the starting and central point of reference, and that their participation in policy making and interventions may be crucial for success.

## Summary

The selective and limited understanding of what is at stake with child soldiers’ recovery and reintegration in each of our disciplines encouraged us to engage in an interdisciplinary dialogue between children’s rights law, transitional justice and psychosocial approaches. This dialogue has helped us to even better understand the limits and potential of each of our own disciplines. Whereas it has not led to a clear-cut grand design, it surely has identified crucial insights on the way forward beyond a binary distinction between victimhood and perpetrator-hood: participation of former child soldiers themselves in conceptual analysis, policy making and interventions seems difficult to escape from; alternative ways of accountability so as to acknowledge the perpetrator-hood dimension deserver further investigation; and individual, social and societal healing may well be much more interconnected than currently accepted.

## Abbreviations

CRC: Convention on the Rights of the Child
DD(R)R: Disarmament, Demobilisation, (Rehabilitation) and Reintegration
MACR: Minimum Age of Criminal Responsibility
OPAC: Optional Protocol on the Involvement of Children in Armed Conflict

## Footnotes

1 In October 2009, the conference “Rehabilitation, Reintegration and Reconciliation of War-Affected Children” was held in Brussels. Scholars, policy makers and practitioners from all over the world, came together to discuss the theme of war-affected children from the perspective of three connected, and yet distinct, disciplines: children’s rights, psychosocial wellbeing and transitional justice. In September 2013, a follow-up conference was organised in Kampala, by the same organisations and War Child in Uganda: “Children and youth affected by armed conflict: Where to go from here?” (www.kampala2013.ugent.be). More than 150 academics, practitioners and policy makers globally, including a large representation from Southern countries, attended this conference, shared their experiences and knowledge, and discussed the way forward in the field of rehabilitation and reintegration processes of children and adolescents affected by armed conflicts.

2 Whereas the typology of protection, provision and participation rights has its limitations and setbacks [63], it remains a useful didactic device to capture the key objectives of children’s rights.
